# Clinic variation in recruitment metrics, patient characteristics and treatment use in a randomized clinical trial of osteoarthritis management

**DOI:** 10.1186/1471-2474-15-413

**Published:** 2014-12-06

**Authors:** Kelli D Allen, Hayden B Bosworth, Ranee Chatterjee, Cynthia J Coffman, Leonor Corsino, Amy S Jeffreys, Eugene Z Oddone, Catherine Stanwyck, William S Yancy, Rowena J Dolor

**Affiliations:** Health Services Research and Development Service, Durham VA Medical Center, Durham, NC USA; Department of Medicine and Thurston Arthritis Research Center, University of North Carolina, Chapel Hill, NC USA; Department of Medicine, Duke University Medical Center, Durham, NC USA; Center for Aging and Human Development, Duke University, Durham, NC USA; Department of Psychiatry and Behavioral Science, Duke University, Durham, NC USA; Department of Biostatistics and Bioinformatics, Duke University, Durham, NC USA; HSR&D (152), VA Medical Center, 508 Fulton Street, Durham, NC 27705 USA; Division of General Internal Medicine, Duke University Medical Center, 411 West Chapel Hill Street Suite 500, Durham, NC 27701 USA

**Keywords:** Osteoarthritis, Health services, Multicenter study

## Abstract

**Background:**

The Patient and PRovider Interventions for Managing Osteoarthritis (OA) in Primary Care (PRIMO) study is one of the first health services trials targeting OA in a multi-site, primary care network. This multi-site approach is important for assessing generalizability of the interventions. These analyses describe heterogeneity in clinic and patient characteristics, as well as recruitment metrics, across PRIMO study clinics.

**Methods:**

Baseline data were obtained from the PRIMO study, which enrolled n = 537 patients from ten Duke Primary Care practices. The following items were examined across clinics with descriptive statistics: (1) *Practice Characteristics*, including primary care specialty, numbers and specialties of providers, numbers of patients age 55+, urban/rural location and county poverty level; (2) *Recruitment Metrics,* including rates of eligibility, refusal and randomization; (3) *Participants’ Characteristics*, including demographic and clinical data (general and OA-related); and (4) *Participants’ Self-Reported OA Treatment Use*, including pharmacological and non-pharmacological therapies. Intraclass correlation coefficients (ICCs) were computed for participant characteristics and OA treatment use to describe between-clinic variation.

**Results:**

Study clinics varied considerably across all measures, with notable differences in numbers of patients age 55+ (1,507-5,400), urban/rural location (ranging from “rural” to “small city”), and proportion of county households below poverty level (12%-26%). Among all medical records reviewed, 19% of patients were initially eligible (10%-31% across clinics), and among these, 17% were randomized into the study (13%-21% across clinics). There was considerable between-clinic variation, as measured by the ICC (>0.01), for the following patient characteristics and OA treatment use variables: age (means: 60.4-66.1 years), gender (66%-88% female), race (16%-61% non-white), low income status (5%-27%), presence of hip OA (26%-68%), presence both knee and hip OA (23%-61%), physical therapy for knee OA (24%-61%) and hip OA (0%-71%), and use of knee brace with metal supports (0%-18%).

**Conclusions:**

Although PRIMO study sites were part of one primary care practice network in one health care system, clinic and patient characteristics varied considerably, as did OA treatment use. This heterogeneity illustrates the importance of including multiple, diverse sites in trials for knee and hip OA, to enhance the generalizability and evaluate potential for real-world implementation.

**Trial registration:**

Clinical Trial Registration Number: NCT 01435109

**Electronic supplementary material:**

The online version of this article (doi:10.1186/1471-2474-15-413) contains supplementary material, which is available to authorized users.

## Background

Knee and hip osteoarthritis (OA) are among the most common chronic health conditions and leading contributors to pain and disability among adults [1–6]. The prevalence of OA is on the rise, and this trend is expected to continue [7]. For example, recent data from the Framingham Osteoarthritis Study show that over the past 20 years, the prevalence of knee OA approximately tripled in men and almost doubled in women [8]. Therefore, in addition to the substantial toll of OA at the individual level [9], this health problem is a significant burden for healthcare systems [10–12].

Evidence-based guidelines emphasize that adequate management of knee and hip OA requires a combination of behavioral and clinical strategies [13–16]. However, many studies show there is underutilization of core treatment approaches [12, 17–21]. For example, although weight management and physical activity are key components of managing knee and hip OA [13, 15, 16], the majority of adults with OA are overweight and physically inactive [17, 22, 23], and provider recommendations for these behavioral strategies are infrequent [19, 24–26]. With respect to clinical care, several studies have shown low pass rates for OA quality of care indicators, including assessment of pain and function, referrals to other providers (when indicated), appropriate prescribing of pain medications, and general use of non-pharmacological treatments [20, 27–30]. These data all highlight the need for evidence-based interventions to augment the use of behavioral strategies and recommended components of clinical care for patients with knee and hip OA, with the ultimate goal of improving pain, function, and other key outcomes.

The Patient and PRovider Interventions for Managing Osteoarthritis in Primary Care (PRIMO) study is being conducted to address these gaps. Since optimal management of OA is multifactorial in nature, PRIMO is designed to simultaneously test both patient-based and provider-based interventions [31]. The provider-based intervention, which delivers patient-specific treatment recommendations to providers at the point of care, is particularly innovative; only a few studies have examined provider-based programs to enhance management of OA in clinic settings [32–34], and none have been in the U.S., to our knowledge. A particularly important feature of PRIMO is that it is being conducted in multiple primary care clinics. This is important for assessing generalizability of the interventions and for understanding whether patient or clinic characteristics may influence the feasibility and effectiveness in real-world practice.

In this manuscript we focus on describing the variation in patient and practice characteristics across the ten clinics included in PRIMO. There are four specific objectives. First, we report on practice-related and geographic variables across study clinics. Second, we present recruitment data for each study site. Third, we report on participant demographic and clinical characteristics across study sites. Fourth, we describe the use of various OA treatments at baseline across the study sites. Understanding how OA treatment use may differ across sites is important for the proposed study because the provider-based intervention aims to enhance use of guideline-based treatments [13–16]. In addition, these data are important because very little is known about patterns and variability in OA treatment in the primary care setting. Although some studies have described OA-related care in other countries [35–37], data are sparse for the U.S., particularly with respect to non-pharmacological care [38–40].

## Methods

### Overview and study design

Detailed methods of the PRIMO study have been published previously [31]. Here we provide an overview of methods relevant to these analyses. PRIMO is a randomized controlled trial with a 2 × 2 factorial design, in which primary care clinics are randomized to Provider Intervention vs. Control, then patients within those clinics are assigned to Patient Intervention vs. Control. Patients were therefore assigned to one of four study arms: 1.) Patient Intervention Only, 2.) Provider Intervention Only, 3.) Patient Intervention + Provider Intervention and, 4.) Usual Care Control. This research was conducted in compliance with the Helsinki Declaration and was approved by Duke University Medical Center Institutional Review Board (Protocol # Pro00022836). All study participants provided written informed consent prior to study participation.

### Study setting

This study is being conducted in ten clinics from the Duke Primary Care Research Consortium, a practice-based research network composed of 30 practices in eight counties of North Carolina. The PRIMO sites were selected to encompass a range of clinics in terms of primary care specialty (Family Medicine and Internal Medicine), patient panel size, number and types of providers, and urban/rural locations. We matched pairs of clinics based on these characteristics and randomized one of each pair to Provider Intervention vs. Provider Control.

### Participants

We aimed to enroll 56 patients per clinic, for a total sample size of n = 560. However, as shown in Table 1, we were unable to meet this recruitment goal at two clinics, and we enrolled 1 additional patient at 2 clinics because of overscheduling. Inclusion criteria were: diagnosis of knee or hip OA (based on physician interpretation of radiographic or magnetic resonance imaging, - documented in the Duke electronic medical records/for either joint OR meeting American College of Rheumatology clinical criteria for knee OA [41]), current symptoms in the joint(s) with OA, body mass index (BMI) ≥ 25, and not currently meeting Departments of Health and Human Services physical activity recommendations [42]. Key exclusion criteria were: other rheumatologic conditions, hip or knee surgery or acute meniscus or anterior cruciate ligament tear in the past six months, recent hospitalization for cardiovascular/cerebrovascular event, serious mental health conditions, on waiting list for hip or knee arthroplasty, motor neuron diseases, terminal illness, and current participation in another OA intervention or lifestyle changes study. A detailed list of exclusion criteria is available elsewhere [31].

**Table 1:** **Characteristics of study clinics**

	Clinic 1	Clinic 2	Clinic 3	Clinic 4	Clinic 5	Clinic 6	Clinic 7	Clinic 8	Clinic 9	Clinic 10	Average
Family (FM) or Internal Medicine (IM)	FM	FM	FM	IM	FM	IM	IM	IM	FM	FM	N/A
# Medical Doctors and Doctors of Osteopathy	6	3	6	8	5	8	8	7	9	6	6.4
# Nurse Practitioners and Physician Assistants	1	4	0	0	2	0	0	0	2	1	1.1
# Patients Age 55+	2,730	5,400	2,865	4,859	2,720	4,590	4,259	2,916	3,817	1,507	3,566
Urban/Rural Setting*	Rural	Rural	Rural	Sm City	Rural	Sm City	Sm City	Lg City	Sm City	Town	N/A
% Households Below Poverty Level in County	19.9%	25.8%	16.4%	19.3%	16.4%	19.3%	19.3%	11.6%	19.3%	16.4%	13.5%

*Lg (large) city = population >250,000; Sm (small) city = population 100,000-250,000; Town = population 20,000-99,999; Rural = population <20,000.

### Recruitment procedures

All primary care providers at each PRIMO clinic were approached regarding participation. Providers were given a summary of the study, and signed a consent form if willing to participate. Most providers participated, but two providers at one clinic and one provider at another clinic declined participation. We used Duke electronic medical records to identify patients of participating providers who had ICD-9 codes for knee/hip OA (715.xx) and knee/hip pain (719.xx); in three clinics (#1, #7, and #10 in Table 1) we also expanded the codes to include pain in a limb (729.5) because we did not meet enrollment goals with the first two sets of codes. We also initially restricted this search to patients who had a visit at study clinics within the past 12 months, to help identify those who were regular patients at these sites; for eight clinics (all but clinics #5 and #9 in Table 1) we then extended this window to 18 months in order to meet enrollment goals. Following these data pulls, we further examined patients’ medical records to confirm the presence of an OA diagnosis (based on criteria noted above) and to check for exclusion criteria. We mailed introductory letters, signed by patients’ primary care providers, to those who met criteria based on the electronic medical record, followed by a screening telephone call to further assess eligibility. Patients who met those screening criteria were then asked to meet a study team member at their clinic site, where we assessed clinical criteria for knee OA (when appropriate) [41], as well as height and weight to determine BMI. Patients who were not overweight or did not meet clinical criteria for knee OA (and also did not have radiographic evidence of knee or hip OA in at least one joint) were excluded from the study.

### Interventions

#### Patient intervention

This is a twelve-month intervention that focuses on three key behaviors important for managing OA [13, 15, 16, 43]: physical activity, weight management, and cognitive behavioral pain management skills. The intervention is delivered via telephone by a health educator, with calls scheduled twice per month for the first six months, then monthly for the last six months. The intervention delivers educational content and emphasizes goal-setting and action planning regarding target behaviors. During the first three months of the study, participants are asked to choose to focus on either weight management or physical activity; the other topic is covered for the second three months. During the final six months of the intervention, participants continue to work on goals related to physical activity and weight management. Cognitive behavioral pain management skills are taught and reinforced throughout the intervention. Participants also receive written educational materials, an OA exercise video and accompanying therapy band, and a CD of relaxation exercises.

#### Provider intervention

This intervention involves delivery of patient-specific OA treatment recommendations, based on published guidelines [14–16, 44], delivered at the point of care. The recommendations include both non-pharmacological and pharmacological therapies [31]. The study team and other content experts developed algorithms to guide which treatment recommendations would be appropriate for a given patient. Clinical data that feeds into these algorithms are derived from medical records and baseline assessments with each participant. The patient-specific treatment recommendations are delivered to primary care providers prior to participants’ first routine (non-urgent) visit after enrolling in the study. Recommendations are delivered to providers within the electronic medical record system.

### Measures

All measures for these analyses were obtained as part of the recruitment process and baseline assessments.

*Clinic Data* included primary care specialty (Family or Internal Medicine), numbers of physicians (medical doctor (MD), doctor of osteopathy (DO)) and mid-level providers (e.g., physician assistant (PA), nurse practitioner (NP)) at the time enrollment began at the clinic, number of patients age 55 years or older (since this is a key risk demographic for lower extremity OA [45]) with at least one visit in 2010 (start of the study), geographic setting (large city, small city, town, or rural) based on US Census Bureau definitions [46], and proportion of households in the county with income below poverty level [47].

*Participant Recruitment Data* included, for each site, the numbers of medical records reviewed and the numbers of patients who were: mailed a recruitment letter, not pursued after the recruitment letter was mailed, refused participation prior to randomization, were found to be ineligible prior to randomization, and were randomized.

*Patient Demographic and Clinical Characteristics* included age, gender, race (white vs. non-white), marital status (married/living with partner vs. other), self-reported income status (low income defined as “just meet basic expenses” or “don't have enough to meet basic expenses”), self-reported general health (excellent, very good, good vs. fair, poor), body mass index (BMI), Western Ontario and McMaster Universities Osteoarthritis Index (WOMAC) – a measure of pain, stiffness and function (range of 0–96 with higher scores indicating worse symptoms) [48, 49], presence of knee OA, hip OA or both (determined as described above for inclusion criteria), and self-reported duration of OA symptoms.

*Osteoarthritis Treatment Variables* included self-reported: current use of any pain medication for OA (as well as specific classes including non-steroidal anti-inflammatory drugs (NSAIDs) and opioid and non-opioid analgesics), ever having used a topical cream for OA, ever having a knee or hip joint injection (separately), ever having seen a physical therapist (PT) for knee or hip OA (separately), ever having used any knee brace and use of knee braces with metal supports (if participants had knee OA), ever having used herbs or supplements for OA.

### Analyses

Descriptive statistics were calculated by clinic. Means and standard deviations were calculated for continuous variables and proportions for dichotomous variables. Intraclass correlation coefficients (ICCs) were used to measure between-clinic variation in patient characteristics and OA treatment use. Substantial between-clinic variation is indicative of within-clinic homogeneity or a clustering effect. ICCs were calculated using random effects models [50, 51].

## Results

### Characteristics of study clinics

As shown in Table 1, this study included a diverse set of primary care clinics. Six were Family Medicine and four Internal Medicine clinics, with a range of 3–9 MD or DO providers and a range of 0–4 mid-level providers (NP, PA). The mean number of patients age 55 or older was 3,566, with a range of 1,507-5,400. Clinic locations ranged from rural to large city settings, and proportions of county households with income below poverty level ranged from 12%-26%.

### Recruitment data

**Figure 1:**
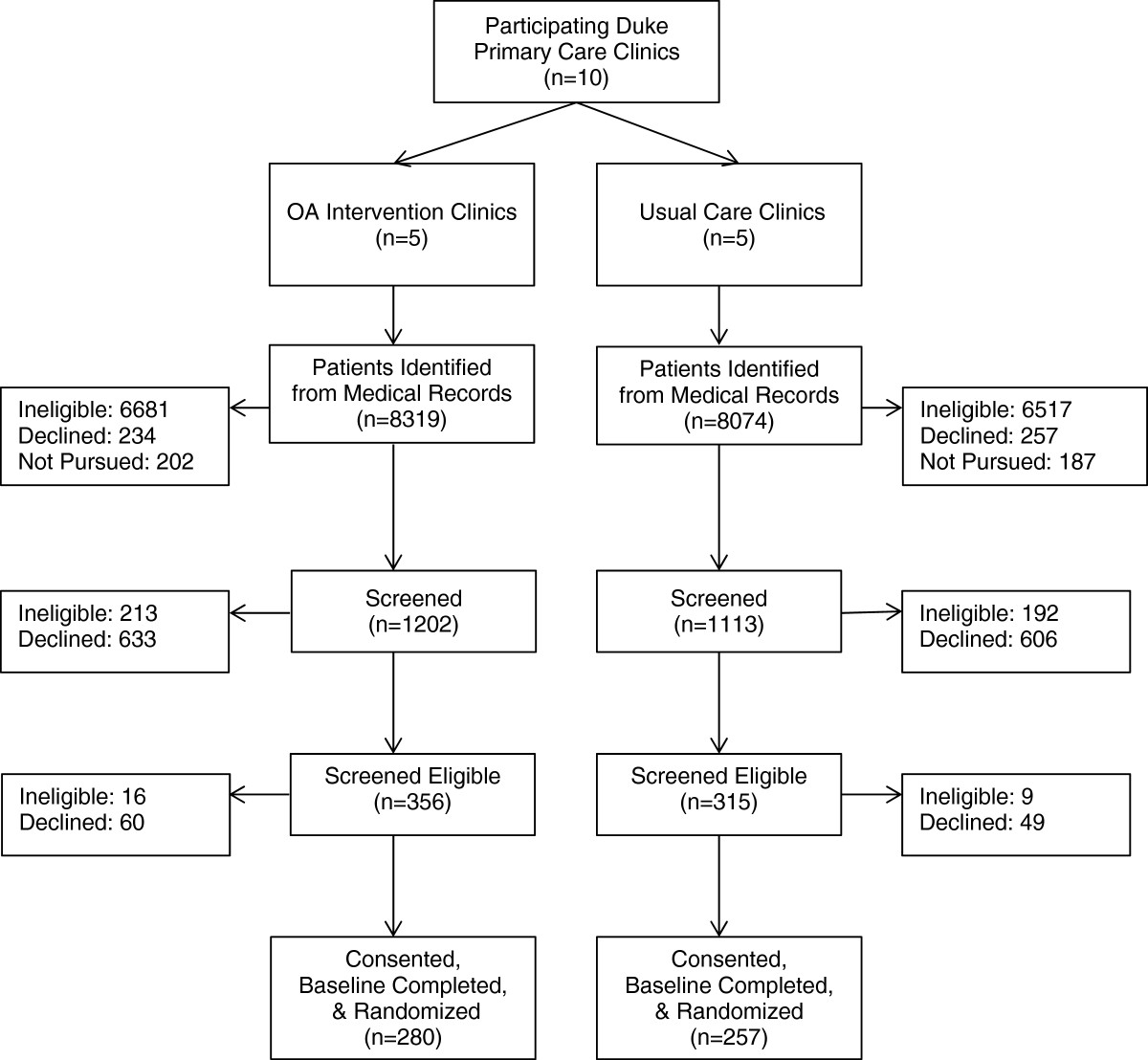
**CONSORT Diagram of flow of participants into the study.**

Figure 1 describes the overall flow of participants into the study. From n = 16,393 individuals initially identified from Duke electronic medical records, based on ICD-9 codes, n = 2,315 were screened, and n = 537 were found to be eligible, agreed to participate in the study and completed baseline assessments. As shown in Table 2, the study team reviewed a large number of medical records to meet enrollment goals for this study. Overall, records of 16,393 potentially eligible patients (based on ICD-9 codes) were reviewed, ranging from 1,185-2,412 across clinics. There were substantial differences across clinics with regard to patients who were eligible based on medical record review; this is illustrated by the proportion of patients who were mailed a recruitment letter after medical record review, which ranged from 10%-31%. Of those who were mailed recruitment letters, 58% refused participation prior to randomization (either by directly notifying the study team or failing to respond after multiple recruitment phone calls), and this ranged from 48%-68% across clinics. An average of 19% of those mailed recruitment letters were subsequently found to be ineligible prior to randomization, and this ranged from 14%-26% across clinics. Overall, 17% of patients who were mailed recruitment letters ultimately were enrolled and randomized into the study, and this ranged from 13%-21% across clinics.

**Table 2:** **Recruitment process variables across ten study clinics**

	Clinic 1	Clinic 2	Clinic 3	Clinic 4	Clinic 5	Clinic 6	Clinic 7	Clinic 8	Clinic 9	Clinic 10	Average
# Medical records reviews	1,532	1,369	1,579	1,883	1,561	1,185	1,638	1,547	1,687	2,412	1,639
# Recruitment letters sent*	319	431	335	355	340	326	307	223	305	233	317
	(21%)	(31%)	(21%)	(19%)	(22%)	(28%)	(19%)	(14%)	(18%)	(10%)	(19%)
# Patients refused before randomization^†^	210	294	190	210	163	178	160	141	157	136	184
	(65%)	(68%)	(56%)	(59%)	(48%)	(55%)	(52%)	(63%)	(52%)	(58%)	(58%)
# Patients ineligible before randomization^†^	43	73	63	63	62	80	79	35	52	57	61
	(14%)	(17%)	(19%)	(18%)	(18%)	(26%)	(26%)	(16%)	(17%)	(25%)	(19%)
# Patients not pursued after letter sent^† ‡^	10	8	26	26	58	11	12	0	40	0	19
	(3%)	(2%)	(8%)	(7%)	(17%)	(3%)	(4%)	(0%)	(13%)	(0%)	(6%)
# Patients randomized^†^	56	56	56	56	57	57	56	47	56	40	54
	(18%)	(13%)	(17%)	(16%)	(17%)	(18%)	(18%)	(21%)	(18%)	(17%)	(17%)

*Percent of medical records reviewed. ^†^Percent of patients who were mailed a recruitment letter. ^‡^Due to enrollment goal being met.

### Participant demographic and clinical characteristics

**Figure 2:**
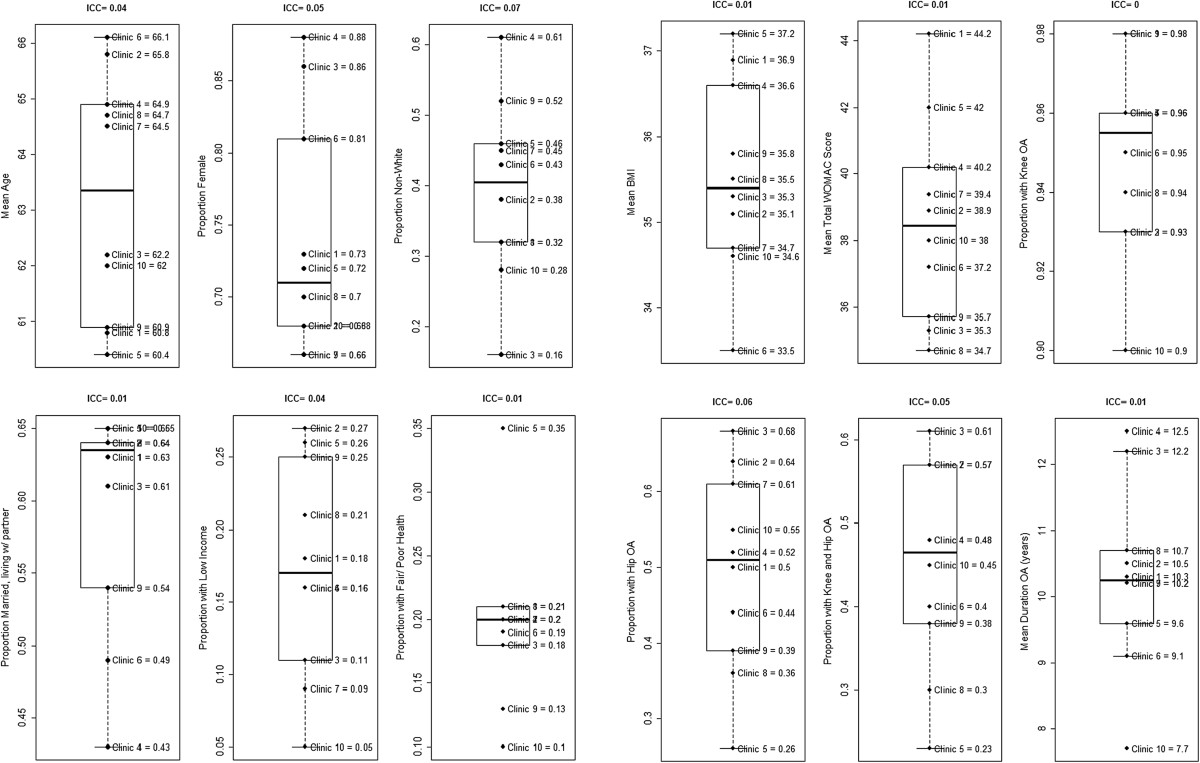
**Participant demographic and clinical characteristics by study clinic.** Notes: Missing data for Race (n = 2), BMI (n = 1), WOMAC (n = 1), Duration of OA Symptoms (n = 1).

Table 3 describes participant demographic and clinical characteristics for the study sample overall. The mean age of participants was over 60, and these ranged from 60.4 to 66.1 years (Table 3 and Figure 2). The proportion of female participants ranged from 66%-88%. Proportions of married patients ranged from 43%-65%, and the proportions with self-reported low income spanned 5%-27%. In terms of clinical and health-related characteristics, 10%-35% of participants reported having “fair” or “poor” overall health, and the mean BMI ranged from 33.5-37.2 kg/m^2^. The mean WOMAC score across clinics was 38.6, which indicates mild to moderate OA symptoms; scores ranged from 34.7-44.2 across clinics. Most patients at all clinics (90%-98%) had a diagnosis of knee OA, 26%-68% had a diagnosis of hip OA, and 23%-61% had both diagnoses. The mean duration of self-reported arthritis symptoms ranged from 7.7-12.5 years.ICCs indicated considerable between-clinic variation (>0.01) for gender, age, race, low income status, hip OA diagnosis, and combined knee and hip OA diagnoses (see Figure 2). There was lower, but potentially meaningful between-clinic variation (ICCs ≤ 0.01) for marital status, BMI, overall health status, WOMAC score, and duration of OA symptoms. There was no estimable between-clinic variation in diagnosis of knee OA (ICC = 0), indicative of no clustering effect for knee OA diagnosis.

**Table 3:** Participant characteristics and self-reported OA treatment use: total sample*

	Mean (SD) or %
Mean age (SD)	63.2 (9.6)
% Female	73.9
% Non-white	39.6
% Married, living with partner	58.9
% Low income	17.7
% with Fair/Poor self rated health	19.9
Mean BMI (SD)	35.6 (7.4)
Mean WOMAC (SD)	38.6 (17.0)
Joints with OA	
% with Knee OA	95.2
% with Hip OA	49.5
% with Knee & Hip OA	44.9
Mean Duration of Arthritis Symptoms (SD)	10.4 (9.2)
Medications for OA	
% Using any pain medication	82.1
% Using analgesic	23.7
% Using NSAID	56.2
% Using opioid	13.0
% ever used topical creams for OA	57.0
% ever had knee joint injection^*^	55.4
% ever had hip joint injection^†^	19.4
% ever seen physical therapist for knee arthritis^*^	39.1
% ever seen physical therapist for hip arthritis^†^	34.0
% ever used knee brace^*^	52.9
% ever used knee brace with metal supports^^^	22.6
% used herbs / supplements	19.7

*From among participants with knee OA; ^†^From among participants with hip OA. ^Among patients who have knee OA and reported using a knee brace.

2 patients were missing race data,,1 patient was missing the WOMAC, 1 patient was missing the duration of arthritis symptoms, and 2 patients were missing data on topical creams.

### OA treatment Use

**Figure 3:**
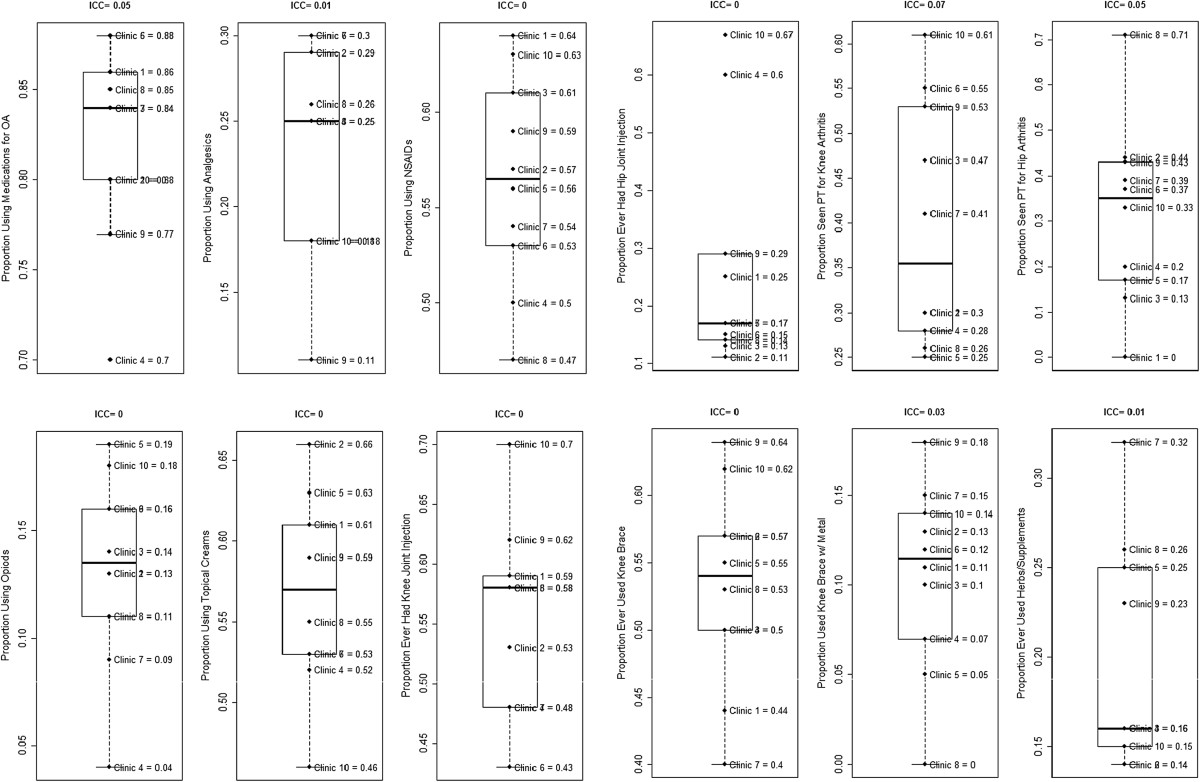
**Self-reported OA treatment use by study clinic.** Notes: Items on physical therapy, joint injections, and knee braces asked only of participants with affected joints. Missing data for Topical Creams (n = 3) and Herbal Supplements (n = 1).

Table 3 describes participants’ self-reported OA treatment use for the study sample overall. The majority of patients at all clinics reported current use of some type of medication to manage their OA symptoms (70%-88%; Table 3 and Figure 3). Use of specific medication classes ranged considerably. NSAIDs were used by 47%-64% of patients, opioid analgesics were used by 4%-19%, and other analgesics were used by 11%-30%. Many patients also reported ever having used topical creams for their OA (46%-66%). Knee joint injections (ever) were reported by 43%-70% of participants with knee OA, and hip joint injections by 11%-67% of participants with hip OA. There was a wide range in proportions of patients with knee OA who reported ever seeing a physical therapist (25%-61%), as well as proportions of those with hip OA who had seen a physical therapist (0%-71%). Among those with knee OA, 40%-64% reported using some type of brace, but only 0%-18% reported ever using a brace with metal supports. Overall, 20% reported using herbs or supplements to treat their OA, and this ranged from 14%-32% across clinics.ICCs indicated considerable between-clinic variation for use of any pain medication (including all classes), PT for knee OA, PT for hip OA, and braces with metal supports for knee OA (see Figure 3). There was lower but potentially meaningful between-clinic variation (ICCs ≤ 0.01) for use of non-opioid analgesics and herbs or supplements. There was no estimable between-clinic variation in use of NSAIDS, opioids, topical creams, joint injections for knee and/or hip, or knee brace (ICC = 0), indicative of potentially no clustering effect for these treatment uses.

## Discussion

A novel and important component of the PRIMO study is the inclusion of a large number of primary care clinics. To our knowledge this is the first US-based clinical trial to involve a provider-based intervention for OA across multiple primary care sites. The PRIMO study clinics varied in important aspects including practice type (Family vs. Internal Medicine), size, and geographic setting. It is particularly important that the PRIMO study included clinics that varied in terms of urban/rural location and poverty level, as these factors can have an impact on the availability of some components of OA treatment, such as physical therapy services. Indeed, we found that some components of self-reported OA treatment use, particularly physical therapy for knee OA, tended to be lower among the three PRIMO clinics in rural locations (e.g. clinics #1, #2 and #5). This highlights the need for strategies to provide physical therapy services for patients in these underserved areas, since this is a core treatment component for knee OA.

Our data on recruitment processes illustrates the considerable effort involved in enrolling patients for this pragmatic clinical trial. Many clinical trials of behavioral interventions for patients with OA have utilized primarily self-referral mechanisms for enrollment [52–56]. We chose to actively enroll patients, via recruitment letters and telephone calls, because self-referral mechanisms may attract the most motivated patients, which may affect generalizability and overestimate intervention effectiveness when applied in real-world clinical settings. However, the type of active enrollment we utilized was very time and labor intensive. As shown in Table 2, 17% of patients who were invited to participate in the study via letter were randomized into the study.

Our recruitment process also differed from most prior OA clinical trials with respect to verification of diagnoses. Prior studies have typically either conducted de novo imaging to verify an OA diagnosis or relied solely on self-report of OA. The former is often impractical for large health services trials like PRIMO, and because we were delivering OA-specific recommendations to primary care providers, we chose not to rely exclusively on self-reported OA diagnosis, which may not have a high degree of accuracy. Therefore, study team members spent a considerable amount of time reviewing medical records to ensure that patients had a prior OA diagnosis, based on prior imaging (or, for knee OA, physician diagnosis plus meeting clinical criteria at the baseline visit [41]). As shown in Table 2, the study team reviewed over 16,000 medical records, with only 19% meeting initial eligibility criteria. One factor contributing to the high volume of records reviewed was inconsistent coding of OA in the medical record, resulting in the need to review records with a broad range of codes (e.g., “joint pain” instead of “osteoarthritis”) that encompass many other conditions. Some prior studies have used algorithms to identify patients with OA and other rheumatic conditions based on medical record data. Among algorithms used to identify people with OA, the specificity has been lower than would be optimal to rely solely on this information for inclusion on a clinical trial. However, these algorithms could be used as an initial step to identify patients who likely have OA, with additional screening conducted only for those individuals; this may be a more efficient approach that reduces the time required for medical record review [57]. More reliable coding of OA in health care settings would also have a tremendous positive impact on researchers’ ability to efficiently identify patients with this health condition for large health services studies.Our recruitment data also illustrate considerable heterogeneity across study clinics. First, there were differences in the proportions of patients who were eligible based on medical record review, ranging from 10%-31%; this was due to differences both in the presence/absence of OA diagnoses and exclusionary health conditions. Second, although the overall randomization rates of those mailed recruitment letters were relatively similar across clinics (13%-21%), there was variability in terms of whether more patients were found to be ineligible (14%-26%) vs. refused participation (48%-68%). As noted above, in order to meet recruitment goals at some clinics, we expanded the list of ICD-9 codes and/or the time window for time of last visit at the clinic. Based on conversations with providers at these clinics, we believe the need to expand the list of ICD-9 codes was largely a reflection of variations in coding practice; some providers tended to use more general joint pain related codes (e.g., 719.xx or 729.5) vs. OA-specific codes (715.xx) regardless of the severity or stage of disease. Although it is possible that use of different code sets across clinics could have led to variation in OA severity we did not see clear evidence of this with respect to baseline WOMAC scores. Specifically, the three clinics at which we expanded to more general codes were not among those with the lowest symptom severity (Figure 3). There were only two clinics in which we did not need to expand the time window since the prior clinic visit from 12 to 18 months. Patients in these two clinics overall may have had more frequent visits to providers, possibly indicating overall poorer health. In one of these clinics (#5), we did find the highest proportion of study participants with “fair or poor” general health. Given the overall heterogeneity in patient health status across clinics, it will be important for future analyses of this study to consider these variations in statistical models.

There was also considerable variability in patient demographic and clinical characteristics across PRIMO clinics, further enhancing generalizability of the study. With respect to demographic characteristics, there was substantial between-clinic variation in age, gender, race, and income status. It is particularly important that this study included clinics with high proportions of non-white patients and those with low income; these two demographic groups have been under-represented in OA clinical trials, and some studies have shown that these groups have lower referrals to and use of some OA-related treatment components [17, 38, 39, 58–60]. Clinic samples also varied in terms of self-reported overall health, with 20%-35% rating their health as fair or poor. This is important because overall health status may impact response to the patient behavioral intervention or the effectiveness of the provider-based intervention. In particular, it may be more challenging for providers to adequately address OA-specific issues for patients who also have multiple comorbid conditions. With regard to OA-specific characteristics, there was not much between-clinic variation in presence of knee OA (≥90% at all clinics), but there was substantial between-clinic variation in diagnosis of hip OA (26%-68%) and diagnosis of both knee and hip OA 23%-61%. Some clinics with a higher prevalence of hip OA were also those in which the average participant age was higher (e.g. clinics #2, #3, and #7). Not surprisingly, the duration of arthritis symptoms was also longer in several of the clinics with the highest average patient age (e.g. clinics 2, 3, and 4). There was between-clinic variation in WOMAC scores, ranging from 34.7-44.2 , on a scale of 0–96; this range likely reflects clinically meaningful differences in average symptom severity across these clinic samples [61].

Finally, we also observed substantial between-clinic variation in some of the self-reported OA treatment use variables. Although the majority of patients at all clinics reported using pain medications for managing OA, there was notable between-clinic variation for use of some specific medication classes. This is not surprising since there is no standard algorithm for pharmacological treatment of OA [13, 15, 62], and patients have differential response to various pain medications. There were also variations in use of non-pharmacological therapies. In particular, there was substantial between-clinic variation in patients who reported ever seeing a physical therapist, both for knee and hip OA. The evidence base for physical therapy as a treatment for knee OA is well established [13–16], but less than 1/3 of patients at half of PRIMO study clinics reported ever having received this treatment, despite the fact that average symptom durations were 8–13 years. In addition, although 40%-64% of patients with knee OA reported using some type of knee brace, much smaller proportions (0%-18%) reported using a more supportive type of brace with metal supports. Although there is some conflicting evidence regarding the effectiveness of braces for knee OA [63], these results may reflect underutilization of this treatment option. We did observe a tendency for patients at some clinics to report greater use across different categories of non-pharmacological treatments. For example, clinics #9 and #10 had among the highest proportions of patients reporting receipt of physical therapy and knee braces (both in general and with metal supports), as well as receipt of knee and hip injections. Interestingly, these two clinics also had the lowest proportions of patients reporting fair or poor health, suggesting that OA-specific treatments may be more highly utilized among patients with fewer competing health problems. Overall, PRIMO data reflect significant variation in the use of OA therapies – particularly non-pharmacological ones - across different primary care settings. This may be partly due to the lack of practical guidance in existing OA treatment recommendations for when particular treatments are best utilized [64].

There are several limitations to this study. First, all data were self-reported, and there may be errors in patients’ recall of past OA treatment use. However, there is not a reliable method for obtaining objective data of this nature, and we have no reason to suspect that recall bias would be differential across clinic sites. Second, although we believe the practices chosen for this study are representative of broader primary care practices in the study region, all practices were part of one health care organization, and it is possible that these clinics, their patient populations and their patterns of OA treatment may differ from other health care settings. Similarly, the recruitment-related data describe our experiences with this particular clinical trial, and other studies may differ in rates of patient eligibility, enrollment and other process variables. Third, we described the number and types of providers at the time enrollment began at each clinic, and in some cases there may have been subsequent personnel changes. However, we know of no large changes in numbers of providers at PRIMO clinics during the study period. Fourth, although we asked participants whether they had ever received physical therapy care for knee or hip OA, we did not assess whether participants had received exercise instructions from other providers. Therefore we may have underestimated the proportions of patients who received some exercise-related recommendations, though other research suggests this type of patient education may be infrequent in primary care [26]. Fifth, in this pragmatic, effectiveness study we did not obtain new radiographs or MRIs to ascertain OA diagnoses but rather recruited patients who had already received these OA diagnoses as part of routine clinical care. Therefore specific criteria used to diagnose OA based on imaging may have differed across clinicians. Sixth, because OA is not always documented in the medical record, some patients in these Duke healthcare system clinics with hip or knee OA may have been inadvertently excluded from potential contact for this study.

## Conclusions

In summary, these data show both the challenges and important advantages of conducting practice-based research, particularly including diverse primary care clinics. Although PRIMO study clinics were part of one health care system, they varied considerably, as did participants enrolled in the study. It is particularly important that we were able to enroll participants that differed in terms of overall health status, income, OA symptom severity, and baseline OA treatment use, as these may all impact intervention response. Inclusion of differing clinics will also allow assessment of practical aspects of implementing a provider-based OA intervention across different primary care settings. Finally, these results illustrate specific challenges of conducting pragmatic trials that actively recruit patients based on electronic medical record data. Two main challenges were variability in diagnostic coding and the volume of time required to review medical records for eligibility criteria. Although this study focused on OA, these challenges would likely apply to many other chronic health problems since coding variability exists for many conditions [65–68]. The increased availability of electronic medical records holds great promise for clinical and health services research, but there is a need for more efficient and standardized processes that facilitate identification and recruitment of patients for these kinds of pragmatic studies.

## Authors’ original submitted files for images

Below are the links to the authors’ original submitted files for images. Authors’ original file for figure 1 Authors’ original file for figure 2 Authors’ original file for figure 3

## Abbreviations

OA: Osteoarthritis
PRIMO: Patient and provider interventions for managing osteoarthritis in primary care
BMI: Body mass index
MD: Medical doctor
DO: Doctor of osteopathy
PA: Physician assistant
NP: Nurse practitioner
WOMAC: Western Ontario and McMaster Universities Osteoarthritis Index
NSAIDs: Nonsteroidal anti-inflammatory drugs
ICCs: Intraclass correlation coefficients
PT: Physical therapy.
