# Supplementary figures and images for: Clinic variation in recruitment metrics, patient characteristics and treatment use in a randomized clinical trial of osteoarthritis management
Source: BMC Musculoskelet Disord. 2014 Dec 6;15:413. doi: 10.1186/1471-2474-15-413 (PMC4295303; doi:10.1186/1471-2474-15-413)

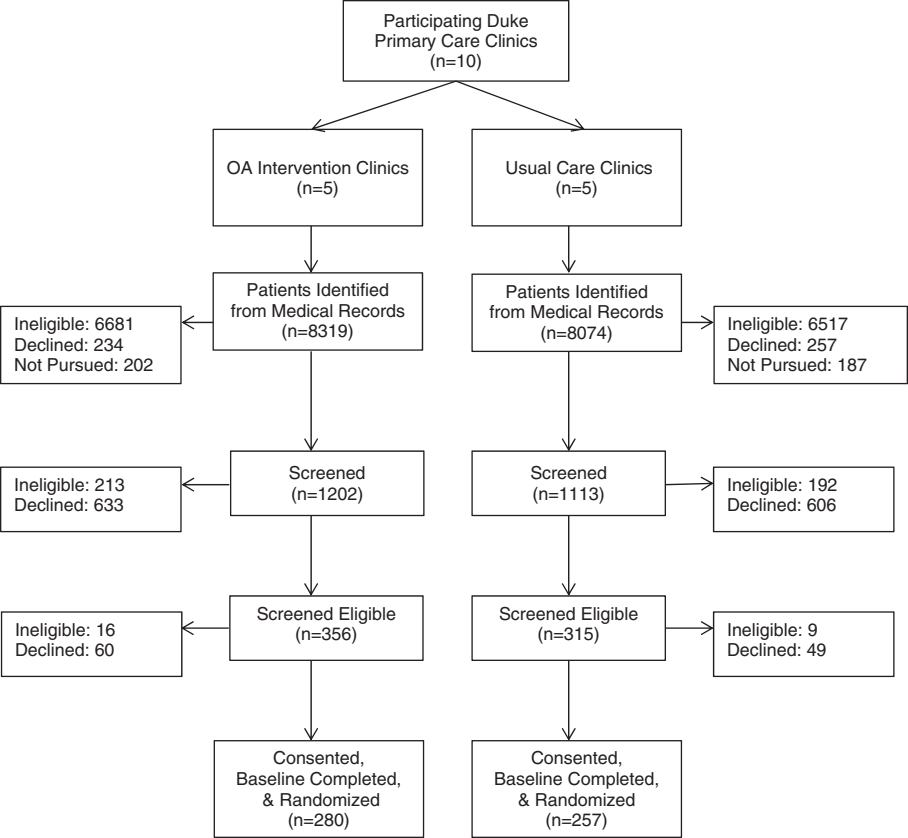

Supplement: Supplementary file 1 — Authors’ original file for figure 1 [file 12891_2014_2357_MOESM1_ESM.pdf]

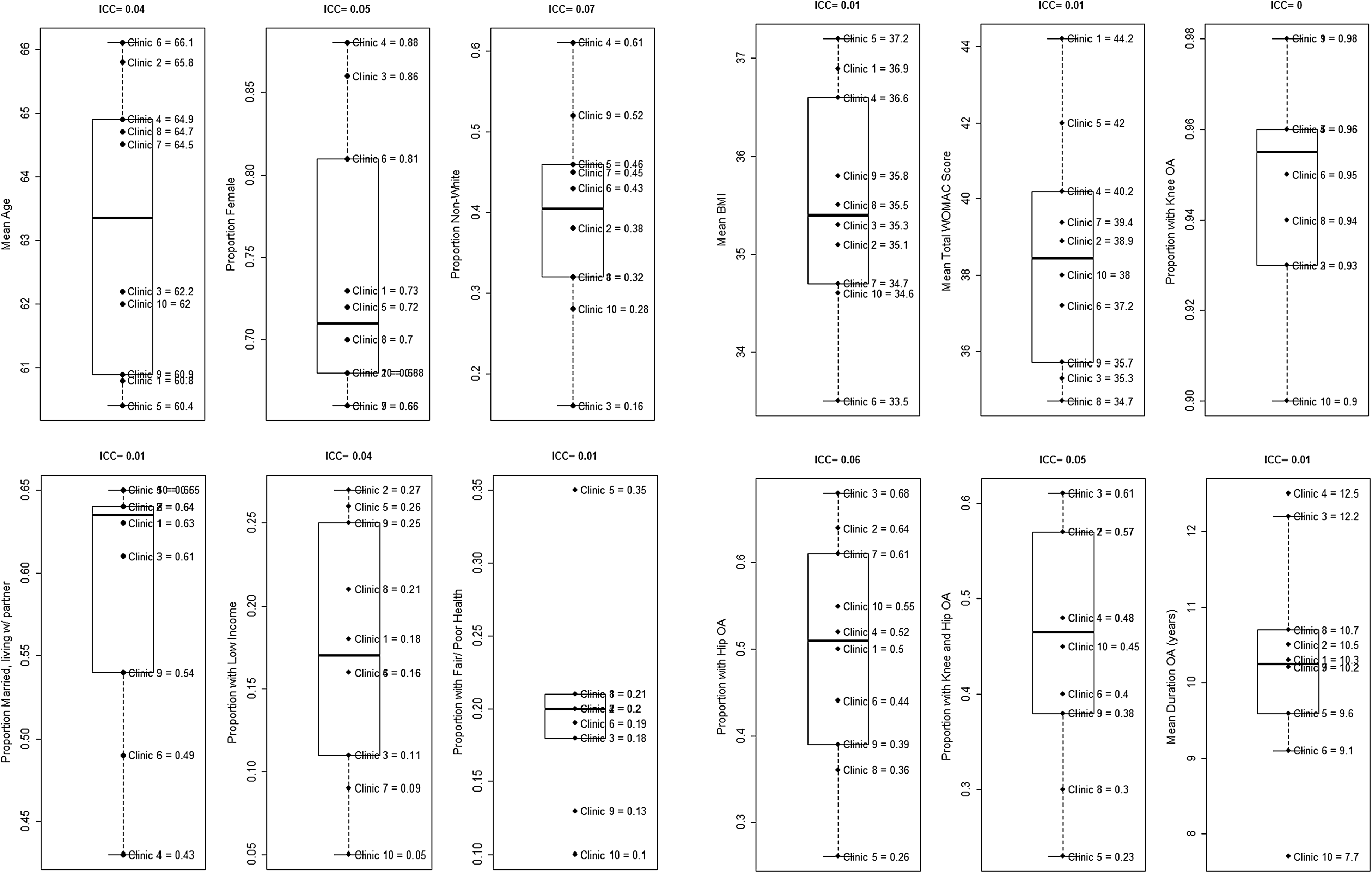

Supplement: Supplementary file 2 — Authors’ original file for figure 2 [file 12891_2014_2357_MOESM2_ESM.tiff]

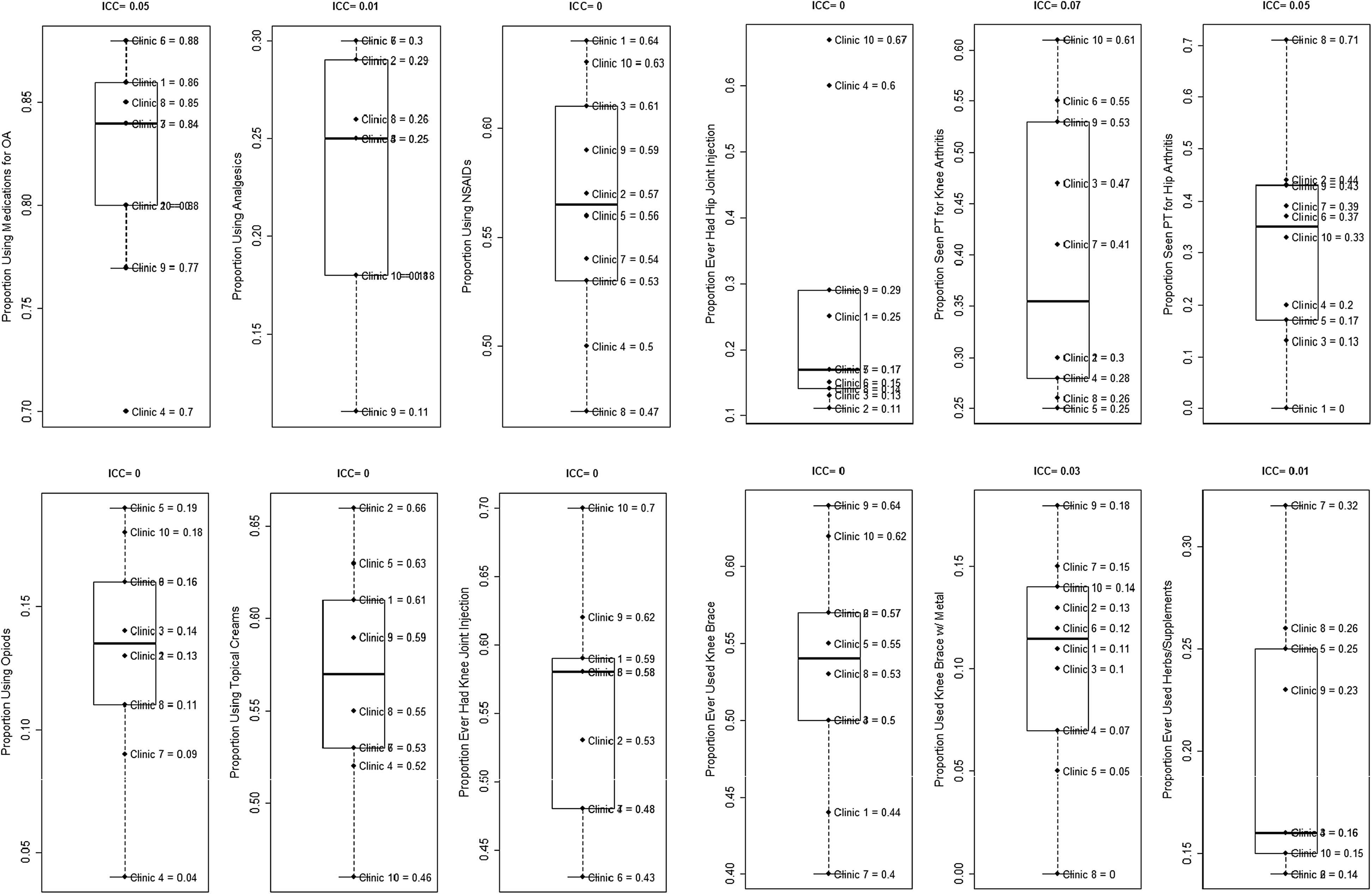

Supplement: Supplementary file 3 — Authors’ original file for figure 3 [file 12891_2014_2357_MOESM3_ESM.tiff]
